# Identification of Biological Markers of Liver X Receptor (LXR) Activation at the Cell Surface of Human Monocytes

**DOI:** 10.1371/journal.pone.0048738

**Published:** 2012-11-21

**Authors:** Cédric Rébé, Rodolphe Filomenko, Magalie Raveneau, Angélique Chevriaux, Minako Ishibashi, Laurent Lagrost, Jean Louis Junien, Philippe Gambert, David Masson

**Affiliations:** 1 Centre de Recherche Institut National de la Santé et de la Recherche Médicale Unité Mixte de Recherche 866, Université de Bourgogne, Dijon, France; 2 Structure Fédérative de Recherche Santé-Sciences et Techniques de l'Information et de la Communication, Université de Bourgogne, Dijon, France; 3 Centre Georges-François Leclerc, Dijon, France; 4 Centre Hospitalier Universitaire Dijon, Dijon, France; Fundação Oswaldo Cruz, Brazil

## Abstract

**Background:**

Liver X receptor (LXR) α and LXR β (NR1H3 and NR1H2) are oxysterol-activated nuclear receptors involved in the control of major metabolic pathways such as cholesterol homeostasis, lipogenesis, inflammation and innate immunity. Synthetic LXR agonists are currently under development and could find applications in various fields such as cardiovascular diseases, cancer, diabetes and neurodegenerative diseases. The clinical development of LXR agonists requires the identification of biological markers for pharmacodynamic studies. In this context, monocytes represent an attractive target to monitor LXR activation. They are easily accessible cells present in peripheral blood; they express LXR α and β and respond to LXR agonist stimulation *in vitro*. The aim of our study was to identify cell surface markers of LXR agonists on monocytes. For this, we focused on clusters of differentiation (CD) markers because they are well characterized and accessible cell surface molecules allowing easy immuno-phenotyping.

**Methodology/Principal Findings:**

By using microarray analysis of monocytes treated or not with an LXR agonist *in vitro*, we selected three CD, *i.e.* CD82, CD226, CD244 for further analysis by real time PCR and flow cytometry. The three CD were up-regulated by LXR agonist treatment *in vitro* in a time- and dose- dependent manner and this induction was LXR specific as assessed by a SiRNA or LXR antagonist strategy. By using flow cytometry, we could demonstrate that the expression of these molecules at the cell surface of monocytes was significantly increased after LXR agonist treatment.

**Conclusions/Significance:**

We have identified three new cell surface markers that could be useful to monitor LXR activation. Future studies will be required to confirm the biological and diagnostic significance of the markers.

## Introduction

Liver X receptor (LXR) α and LXR β (NR1H3 and NR1H2 respectively) are two nuclear receptors activated by oxysterols. LXR α is expressed mainly in the liver, intestine, adipose tissue and macrophages, in contrast LXR β is ubiquitously expressed [1]. During the last 20 years, numerous studies have demonstrated that LXRs are involved in the control of major metabolic pathways such as cholesterol homeostasis, lipogenesis, inflammation and innate immunity, underlying the potential of LXR modulation in human therapeutic [2], [3]. Pharmacological LXR agonists are currently under development and could find applications in various fields such as cardiovascular diseases, cancer, diabetes and Alzheimer disease [4]. One major potential application for LXR agonists is the prevention and treatment of atherosclerosis. Indeed, LXRs have the ability to prevent foam cell formation and to stimulate the elimination of cholesterol from the body via the reverse cholesterol transport (RCT) pathway [5], [6]. LXR target genes are present at all steps of RCT including cellular cholesterol efflux (ABCA1, ABCG1 and Apolipoprotein E), plasma lipid transport (cholesteryl ester transfer protein and phospholipid transfer protein) and biliary cholesterol and bile acid excretion (ABCG5/G8 and CYP7A1) [7]–[12]. *In vivo* studies have clearly confirmed that LXR agonists are atheroprotective in different animal models while LXR deficiency is associated with accelerated atherosclerosis in mice [13]–[16]. Selective depletion of LXR α and β in bone marrow cells increases atherosclerosis development in ApoE and Ldlr−/− mice while pharmacological LXR agonists had a reduced impact in the absence of LXR in the haematopoietic cells [17], [18]. In contrast, selective depletion of LXR α in the liver does not affect the atheroprotective potential of LXR agonists [19]. These data suggest therefore that macrophages/foam cells are the primary targets for LXR agonists in the context of atherosclerosis prevention or treatment.

The clinical development of LXR agonists requires the identification of biological markers to assess the activity of the molecules *in vivo*. First studies in the mouse suggested that changes in lipoprotein profile could be useful to monitor LXR activation. Indeed LXR agonists increase HDL cholesterol in a dose dependent manner in the mouse [20]–[22]. However, unlike human, the mouse is a CETP-deficient species and subsequent studies in CETP transgenic mice, rabbits and primates clearly demonstrated that LXR agonists do not increase plasma HDL concentration in the presence of CETP [22]–[24]. This is explained by the CETP-mediated transfer of cholesterol from HDL to apoB-containing lipoproteins. Interestingly, the lack of increase in HDL cholesterol concentration did not affect the ability of LXR agonists to stimulate reverse cholesterol transport from macrophages suggesting that LXR agonists are still atheroprotective in the presence of CETP [6], [22]. In the quest of other easily accessible biomarkers of LXR activity some groups, including our, proposed that peripheral blood monocytes could be useful to monitor LXR activation [25], [26]. Monocytes are produced in the bone marrow by differentiation of myeloid precursors, reside in the blood for few days, and then are recruited in tissues where they undergo differentiation. Thus they are the direct precursors of macrophage foam cells present in atherosclerotic plaques. Monocytes express both LXR α and β and *in vitro* data demonstrate that many genes are regulated in a similar way in monocytes and macrophages [25], [26]. The aim of our study was to identify cell surface markers of LXR agonists in peripheral blood monocytes. For this purpose, we focused on clusters of differentiation (CD) markers because they are well characterized and easily accessible cell surface molecules allowing immuno-phenotyping. By using microarray analysis we observed that the expression of three CD, *i.e.* CD82, CD226, CD244 was up-regulated in monocytes treated with the LXR agonist T0901317 *in vitro* compared with untreated monocytes. We then selected these CD for further analysis by real time PCR and flow cytometry. In this paper, we demonstrate that LXR agonist treatment increases the expression of these molecules at the cell surface of monocytes in a time- and dose dependent manner, making them accessible markers for monitoring treatment with LXR agonists.

## Results

### LXR agonist treatment increases CD82, CD226 and CD244 mRNA levels in human monocytes, macrophages and foam cells

**Figure 1:**
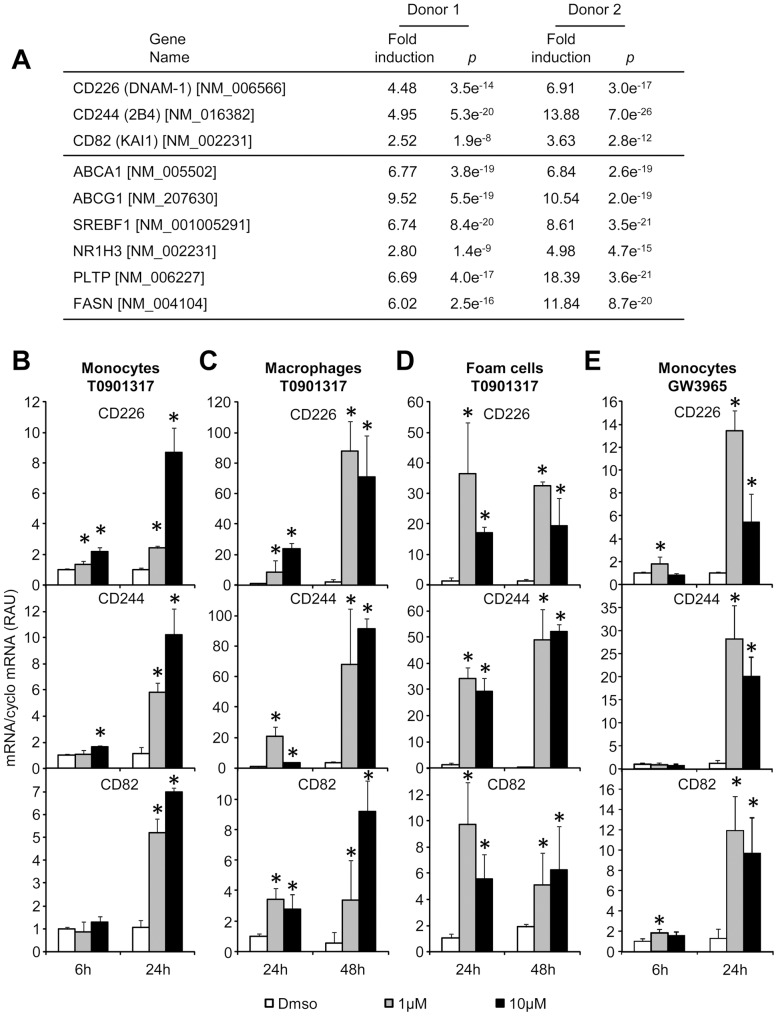
Identification of new LXR targets in human monocytes. **A:** Primary human monocytes were treated 24 hours with 10 µM T0901317 to perform microarray experiments. Fold induction represents gene expression fold increase between untreated monocytes and treated monocytes. *p* indicates the probability value calculation performed in the Rosetta Resolver system. **B to E:** Monocytes (B and E) macrophages (C)or foam cells (D) were treated with indicated concentrations of T0901317 (B, C and D) or GW3965 (E) at indicated times. CD226, CD244 and CD82 mRNA were evaluated by quantitative PCR. Each bar is the mean ± S.D. of triplicates determination of one experiment performed with one healthy donor. Results are representative of 5 independent experiments LXR regulation of CD markers has been validated in five distinct healthy donors * : significantly different from DMSO treatment (P<0.05 Mann-Whitney test).

Human monocytes were isolated from the blood of healthy human volunteers and were treated for 48 hours *in vitro* with the LXR agonist T0901317 at 10 µM or with the solvent only before RNA extraction and microarray analysis. Amongst the CD markers, 3 genes were highly induced by LXR agonist treatment (figure 1A): CD82 (tetraspanin 27 or KAI1), CD226 (DNAX accessory molecule 1) and CD244 (natural killer cell receptor 2B4). mRNA levels of these genes were induced by at least 2.5 fold in the monocytes of two different healthy donors in T0901317 conditions as compared to untreated monocytes. As expected, canonical LXR-target genes such as ABCA1, ABCG1, PLTP or SREBP1c were also highly induced in the same conditions. Real time PCR analysis confirmed the microarray data with a time- and dose-dependent response of the genes to the LXR agonist T0901317 (figure 1B and figure S2). Incubation of cells with the LXR agonist for 6 hours led to only limited changes, however after 24 hours incubation, mRNA levels of the three genes were highly induced as compared to DMSO conditions (figure 1B).

In a second step, we wanted to assess whether increased mRNA levels in monocytes after T0901317 treatment reflect the response of differentiated macrophages or foam cells to LXR agonist stimulation. To this aim, monocytes were differentiated for 6 days in the presence of M-CSF and then loaded or not with cholesterol by 24 hours incubation with acetylated LDL before LXR agonist treatment. As shown in figure 1C and 1D, macrophages and foam cells respond in a similar way as monocytes with marked induction of CD82, CD226 and CD244 after 24 and 48 hours incubations with LXR agonist. The increase after LXR agonist treatment was however much more pronounced for CD226 and CD244 as compared to monocytes, whereas CD82 induction was similar in both cell types. Maximal induction was observed at 1 µM of LXR agonist for 48 hours with no further increase at the 10 µM except for foam cells that present quite similar inductions at 24- and 48- hours of treatment.

Finally, we measured the impact of another LXR specific agonist, GW3965 in monocytes treated for 6 or 24 hours. As shown in figure 1E, GW3965 treatment significantly increased mRNA levels of CD82, CD244, and CD226 after 24 hours with a maximal induction obtained with 1 µM of LXR agonist and no further increase at the 10 µM concentrations. After 6 hours of treatment only, changes in mRNA levels were limited.

### Effect of 22-S-OH cholesterol treatment on CD82, CD226 and CD244 mRNA levels

**Figure 2:**
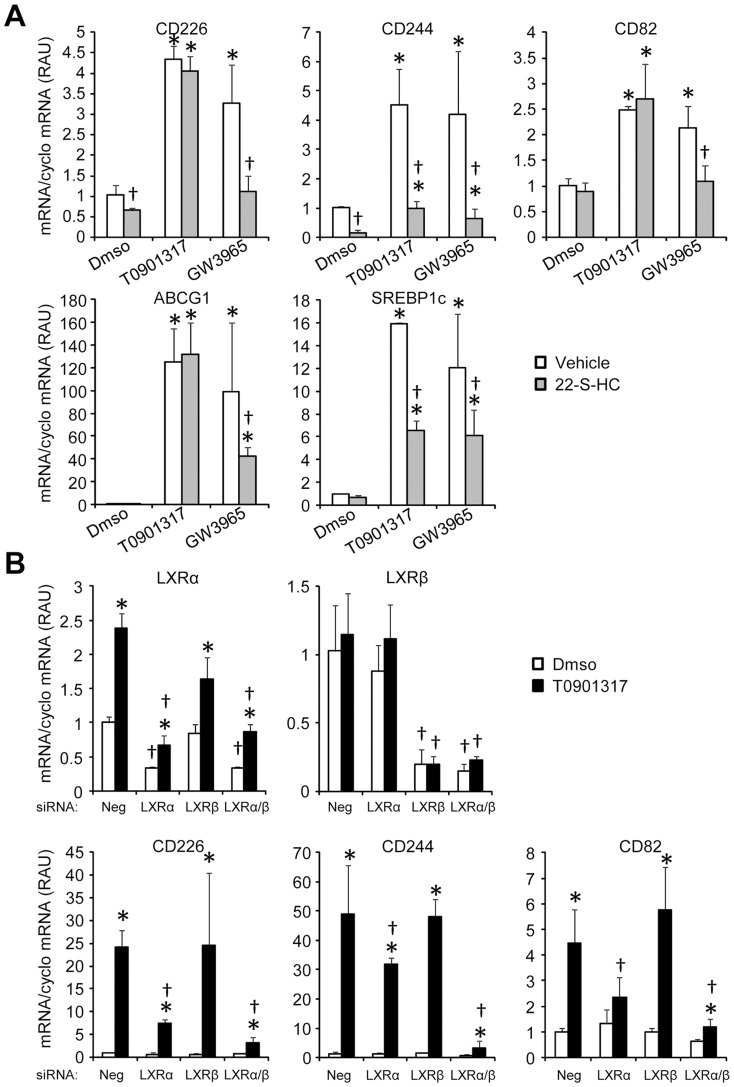
Effect of 22-S-HC on CD82, CD226 and CD244 expression in monocytes treated or not with LXR agonists. **A:** Monocytes were treated with T0901317 or GW3965 at 1 µM in the presence or in the absence of 22-S-HC at 10 µM. CD226, CD244 and CD82 mRNA levels were evaluated by real time PCR. Results are representative of 3 independent experiments. Each bar is the mean ± S.D. of triplicates determination. * : significantly different from DMSO, same treatment, † : significantly different from vehicle conditions (no 22-S-HC) (P<0.05 Mann-Whitney test). **LXR marker regulation by LXR α and β isoforms.** **B:** Monocytes differentiated into macrophages were transiently transfected with either non-targeted siRNA (Neg), or siRNA specific for LXRα, LXRβ, or both and then treated for 24 hours with DMSO or 10 µM T0901317. LXRα, LXRβ, CD226, CD244 and CD82 mRNA expression was evaluated by quantitative PCR. Results are representative of 2 independent experiments. Each bar is the mean ± S.D. of triplicates determination. * : significantly different from DMSO treatment same siRNA conditions; † : significantly different from negative SiRNA same treatment conditions (P<0.05 Mann-Whitney test).

Previous studies have demonstrated that 22-S-Hydroxycholesterol (22-S-HC) behaves as an LXR antagonist [27], [28]. In order to confirm that the increase of CD82, CD226 and CD244 mRNA levels observed after T0901317 and GW3965 treatments was LXR specific, we tested the impact of 22-S-hydroxycholesterol (22-S-HC) on the mRNA levels of the three markers in basal and LXR agonist-treated conditions. We choose to use 22-S-HC at 10 µM and GW3965 or T0901317 at 1 µM as described in previous publications. As shown in figure 2A, 22-S-HC efficiently suppressed the GW3965-mediated induction of the three markers and the T0901317-mediated increase of CD244 mRNA levels. In contrast, 22-S-HC had no effect on CD82 and CD226 mRNA levels in the T0901317 condition. Importantly, CD82, CD226 and CD244 behave as typical LXR targets genes such as ABCG1 or SREBP1c (figure 2A) with a reduction of mRNA levels in the GW3965 conditions for both genes and a significant effect of 22-S-HC in T0901317 conditions for SREBP1c only.

Overall, these results suggest that the three markers are regulated in an LXR-specific way, the lack of effect of 22-S-HC observed for some genes in the T0901317-conditions is likely explained because T0901317 is a stronger LXR activator than GW3965 [29].

### Effect of LXR α and β knock-down on CD82, CD226 and CD244 expression

In further step we determined whether the T0901317-mediated induction of CD82, CD226 and CD244 was dependent on LXR α and/or LXR β. To this aim, we used a LXR silencing strategy in M-CSF differentiated macrophages. As shown in figure 2B, significant knock-down (KD) was obtained for LXR α and β in both DMSO and T0901317-treated conditions. LXR α KD significantly attenuated the T0901317-mediated increase of CD226 and CD82 mRNA levels while a non significant tendency was observed for CD244. In contrast, LXR β KD had no impact on mRNA levels for the three genes. LXR α/β double KD resulted in a reduction of the mRNA levels for CD82 and CD226 similar to what was observed for LXR α KD. Importantly, LXR α/β double KD was able to decrease CD244 mRNA levels in T091317-treated cells. Overall, these results demonstrate that the effects of the agonist T0901317 are specifically mediated by LXR activation. Moreover it suggests that CD226 and CD82 are mainly regulated by LXR α with little or no compensatory effect of LXR β. In contrast, CD244 appears to be regulated by both LXR α and β. Interestingly, CDs behaved as typical LXR target genes such as SREBP1c (figure S3).

### Impact of LXR agonist treatment on the expression of CD226, CD244 and CD82 at the cell surface of monocytes

To determine if the induction of the mRNA levels of the 3 CD markers translates into increased protein levels at the surface of monocytes, expression of CD82, CD226 and CD244 was measured by flow cytometry in isolated monocytes treated for 24 or 48 hours with T0901317 at 10 µM.

**Figure 3:**
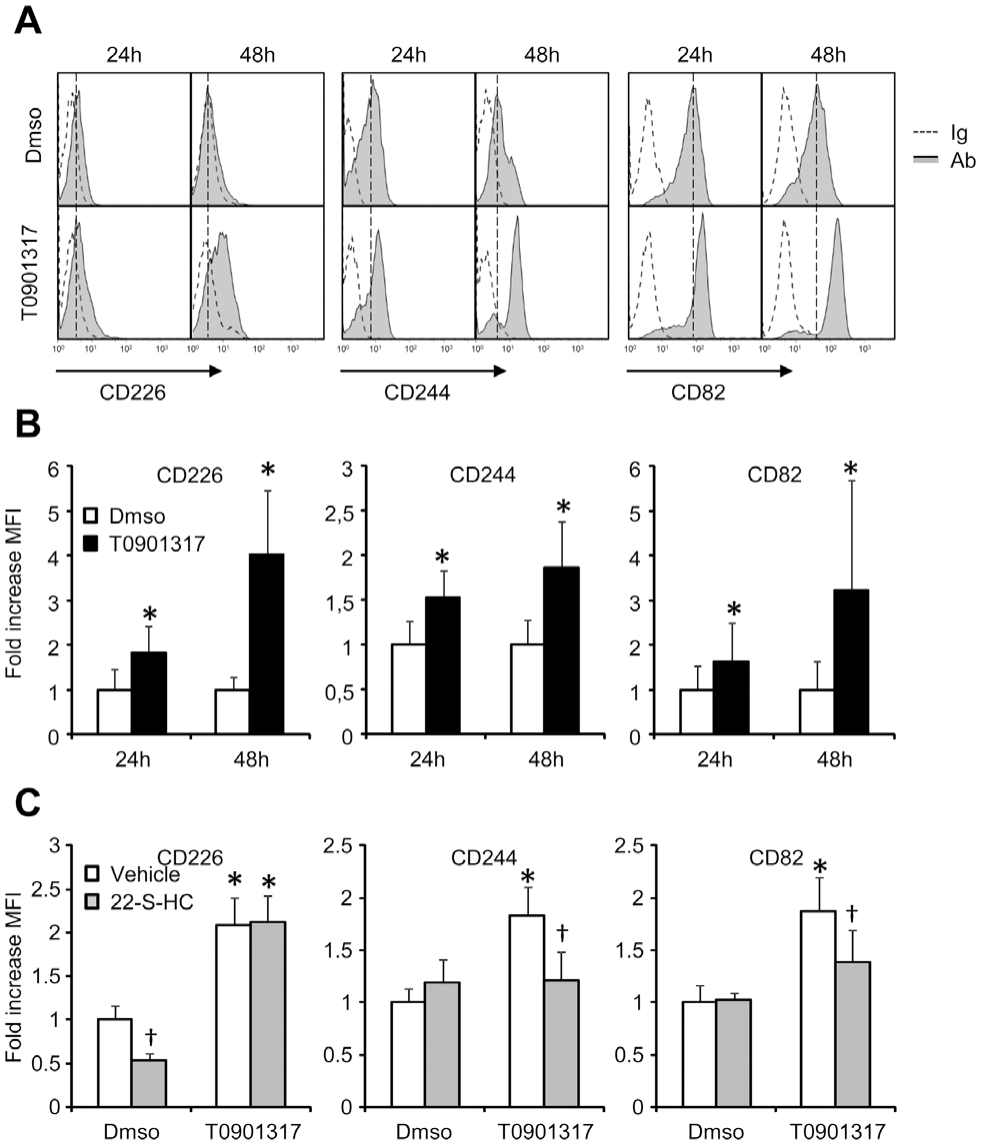
Induction of LXR markers by T0901317 on monocyte surface. Purified monocytes were treated with DMSO or 10 µM T0901317 for 24 or 48 hours and CD226, CD244 or CD82 cell surface expression was evaluated by flow cytometry. **A:** representative histograms. Ig: control immunoglobulin; Ab: specific antibody. **B:** MFI in LXR agonist- and DMSO- treated cells. Each bar is the mean ± S.E.M. of 5 independent experiments using monocytes from 5 distinct healthy donors.. Values are set at 1 in the DMSO conditions. * : significantly different from DMSO treatment (P<0.05 Wilcoxon T test). **C:** MFI in LXR agonist- and DMSO- treated cells with or without 22-S-HC added. Each bar is the mean ± S.E.M. of 3 independent experiments using monocytes from 3 distinct healthy donors.. Values are set at 1 in the DMSO conditions. *: significantly different from DMSO treatment (P<0.05 Wilcoxon T test); † : significantly different from vehicle only conditions (P<0.05 Wilcoxon T test).

As shown in figure 3A, CD226 and CD244 were detected at low levels at the cell surface of monocytes at basal state while CD82 was expressed at a relatively high level. Incubation of the cells with the LXR agonist resulted in a moderate increase of the expression of the three CD (approx. 1.5 to 2 fold increase) while the induction was more pronounced after 48 hours incubation (figure 3B). In these conditions, T0901317 treatment led to an approx. 4 fold increase in the mean fluorescence intensity (MFI) for CD226, 2 fold increase for CD244 and 3 fold increase for CD82. We tested the effect of 22-S-HC treatment on MFI of three CDs in basal and T0901317 conditions. In these new experiments, T0901317 was used at 1 µM and 22-S-HC at 10 µM. Results were consistent with the changes previously observed at the mRNA level. T0901317 at 1 µM significantly increased the MFI of the three CDs. In these conditions, a significant impact of 22-S-HC on the MFI was retrieved for CD244 only (figure 3C). A tendency was also observed for CD82 but did not reach the statistical significance.

### Expression of CD82, CD226 and CD244 in peripheral blood mononuclear cells

**Figure 4:**
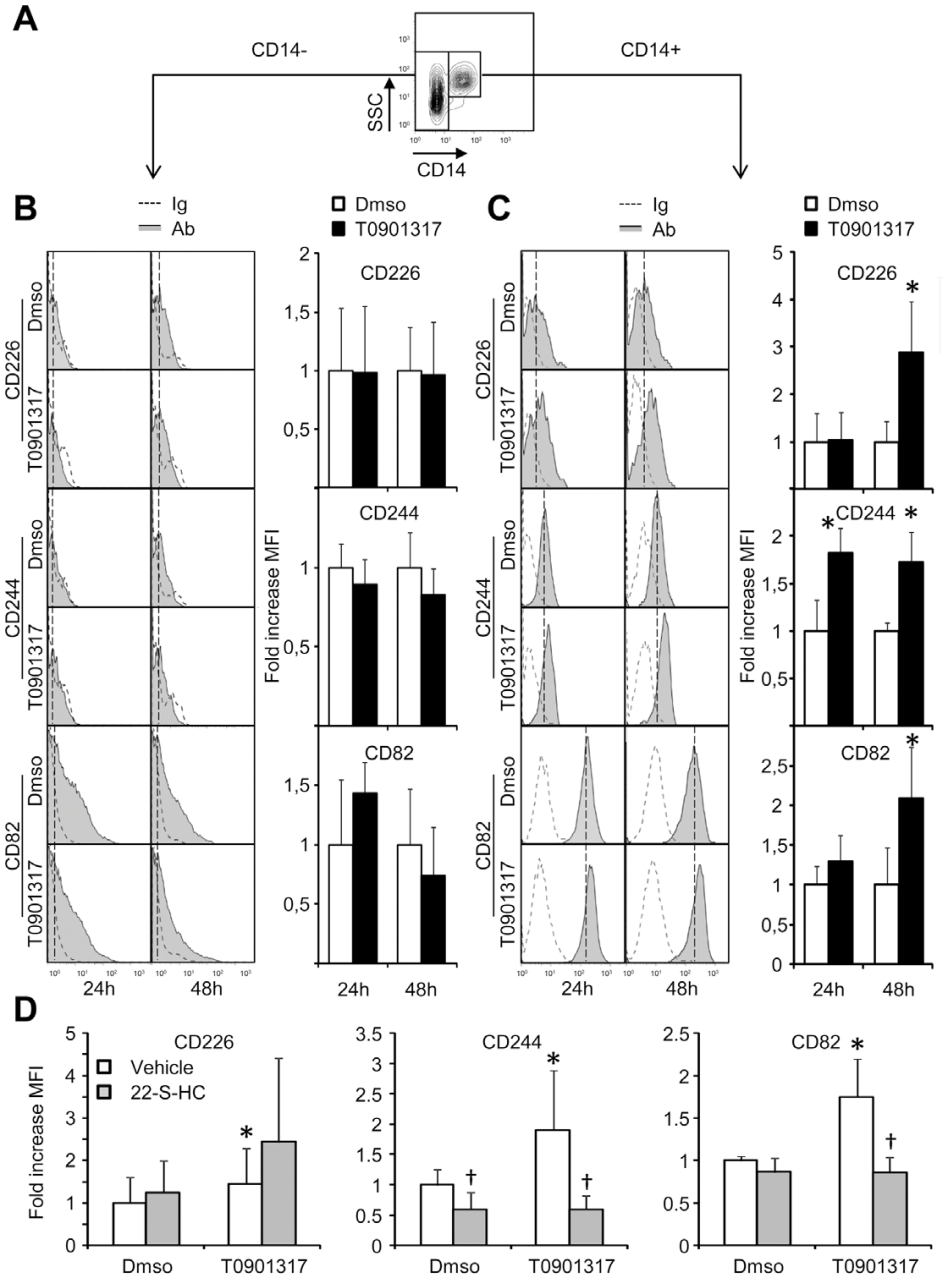
Induction of LXR markers by T0901317 on CD14+ and CD14− cells among PBMCs. Isolated PBMCs were treated with DMSO or 10 µM T0901317 for 24 or 48 hours and CD226, CD244 or CD82 cell surface expression was evaluated by flow cytometry. **A:** Dot plot representing CD14− and CD14+ selected populations. **B:** CD226, CD244 or CD82 on CD14− cells. Right panel, representative histograms. Ig: control immunoglobulin; Ab: specific antibody. Left panel, MFI in LXR agonist- and DMSO- treated cells. Each bar is the mean ± S.E.M. of 4 independent experiments using cells from 4 distinct healthy donors. **C:** CD226, CD244 or CD82 on CD14+ cells. Right panel, representative histograms. Ig: control immunoglobulin; Ab: specific antibody. Left panel, MFI in LXR agonist- and DMSO- treated cells. Each bar is the mean ± S.E.M. of 4 independent experiments. Values are set at 1 in the DMSO conditions. * : significantly different from DMSO treatment (P<0.05 Wilcoxon T test). **D:** MFI in LXR agonist- and DMSO- treated PBMCswith or without 22-S-OH cholesterol added. Each bar is the mean ± S.E.M. of 3 independent experiments using CD14+ gated PBMCs from 3 distinct healthy donors. Values are set at 1 in the DMSO conditions. *: significantly different from DMSO treatment (P<0.05 Wilcoxon T test); †: significantly different from vehicle only conditions (P<0.05 Wilcoxon T test).

Finally, we checked whether we could monitor the induction of LXR targets without selective isolation of monocytes. To this aim, peripheral blood mononuclear cells prepared by ficoll gradient centrifugation were, one day after, treated with the LXR agonist for 24 or 48 hours. Double labelling was performed with an APC-conjugated anti-CD14 antibody and specific FITC-conjugated antibodies against CD82, CD226 or CD244. As shown in figure 4A, monocytes were selected as CD14+ cells. In CD14− negative cells, expression levels of the three CD was barely detectable with almost no positive labelling of the cells as compared to control immunoglobulin (Ig) and there was no significant induction of any of the three CD after LXR agonist treatment (figure 4B). In contrast, in the CD14+ population and as observed previously with isolated monocytes, LXR agonist treatment had little effects on CD expression after 24 hours of treatment (only CD244 expression was slightly increased), but significantly increased the expression of the three CD after 48 h treatment (figure 4C). In CD14+ PBMCs, 22-S-HC treatment significantly decreased both CD244 and CD82 MFI in the T0901317- conditions while no statistically significant effect was observed for CD226.

## Discussion

The aim of our work was to identify new markers of LXR activation at the cell surface of monocytes. We choose to focus on monocytes because they are easily accessible cells from the peripheral blood. They are closely related to macrophages that are present in atherosclerotic plaques. Previous studies have demonstrated that beside LXR β that is ubiquitously expressed, LXR α is present at relatively high levels in monocytes as compared to other blood cells [26]. Accordingly, monocytes respond to LXR agonist treatment in a similar way as macrophages. *In vitro* treatment of monocytes with an LXR agonist result in a dose- and time- dependent induction of LXR target genes such as ABCA1, ABCG1 and RAR α [25], [26]. *In vivo* data in humans are limited but in a recent study, Di Blasio-Smith et al. observed that a single oral dose of LXR agonist induces a significant time-dependent increase of ABCA1 and ABCG1 mRNA levels in the whole blood of an healthy subject [26]. It clearly demonstrates that blood cells, including monocytes, respond to the agonist *in vivo* and can be used for pharmacodynamic studies. However, in this study, mRNA levels of LXR target genes were measured in the whole blood. This requires mRNA extraction, reverse transcription of these mRNA into cDNA and quantitative PCR and therefore the specific response of distinct cell populations to the LXR agonist was not addressed.

In the present study, we have identified 3 CD *i.e.* CD82, CD226 and CD244, induced by agonist treatment at the mRNA level in an LXR specific manner. Their expression was significantly increased at the cell surface of monocytes after LXR agonist treatment and we could monitor the response of the monocytes to LXR agonist treatment by flow cytometry analysis either on isolated cells or among total PBMCs. As we showed, LXR target gene expression was not modulated by T0901317 in non-monocytic cells, raising the importance to evaluate the effect of LXR agonists specifically on monocytes.

The specific function of these three markers in monocytes is not well defined to date. CD82 is a glycoprotein that belongs to the tetraspanin superfamily and is known as a metastasis suppressor [30]. As other tetraspanins, CD82 is involved in the regulation of membrane dynamic and protein trafficking in cell membranes. CD226 (DNAX accessory molecule 1 (DNAM-1)) is a transmembrane glycoprotein involved in T cell and natural killer (NK) cell cytotoxicity. It is a member of the immunoglobulin superfamily and was previously shown to be able to stimulate monocyte extravasation via its interaction with CD155 that is expressed at endothelial cell junctions [31]. CD244 (2B4) is a member of the signaling lymphocyte-activation molecule (SLAM) family that also belongs to the immunoglobulin (Ig) superfamily of molecules [32]. CD244 is expressed in NK cells, some T cells, monocytes and basophils. CD244 is involved in the regulation of NK cell cytotoxicity but its function in monocyte is not characterized [33]. Future studies will be needed to determine whether upregulation of these molecules by LXR agonists affect monocyte/macrophage functions.

There are some specific points of the study we want to discuss. First, LXR mediated-increase of the cell surface markers was less pronounced than the induction at the mRNA level. Nevertheless this induction was still highly significant and allowed us to validate the increase by flow cytometry analysis in the monocytes from 4 different donors in a reproducible way. Second, the induction of the membrane expression of our markers was maximal after 48 hours incubation with LXR agonist but less pronounced after 24 h only, suggesting that these targets could not be used as early markers of LXR activation. However, this sustainability could also be seen as an advantage in comparison to transitory induced LXR target genes. Third, CD226 and CD82 were mainly regulated by LXR α suggesting that specific LXR β activators are unlikely to induce the expression of these two genes. For these ligands, CD244 could be used as bio-marker, because it appears to be significantly regulated by both LXR isoforms. Finally, the markers were only validated through *in vitro* experiments but not *in vivo*. The biological and diagnostic significance of the markers remain therefore to be established. The three CD were however robustly induced by two different LXR agonists in a time- and dose- dependent manner strongly suggesting that significant *in vivo* exposure of blood monocytes to LXR agonists will result in a significant induction of the three markers.

In conclusion, we propose that these three markers could be useful to develop new LXR agonists and to monitor LXR activation in future clinical studies.

## Materials and Methods

### Reagents

Macrophage-Colony Stimulating Factor (M-CSF) was purchased from Miltenyi Biotec, T0901317 from Bertin Pharma. 22-S-HC was a kind gift of Dr. Gerard Lizard. The following FITC conjugated antibodies were used: anti-human CD226, anti-human CD244 (BD Biosciences) and anti-human CD82 (eBioscience). The following APC conjugated antibody was used: anti-human CD14 (BD Biosciences).

### Cell Culture

Human peripheral blood monocytes were obtained from healthy donors with informed consent, provided in accordance with the Declaration of Helsinki by the Etablissement Français du Sang (Besançon, France) and purified as previously described [25]. Briefly mononuclear cells were isolated by Ficoll gradient centrifugation and monocyte negative selection was performed via magnetic activated cell sorting using the Monocyte Isolation Kit II (Miltenyi Biotec) according to the manufacturer's instructions (Figure S1). Monocytes were cultured in RPMI 1640 medium with glutamax-I (Life technologies) supplemented with 10% (vol/vol) fetal bovine serum (FBS; Life technologies) in an atmosphere of 95% air and 5% CO_2_ at 37°C and differentiated into macrophages for 6 days with 100 ng/mL of M-CSF. Ac-LDL loading was performed after 6 days of differentiation with a 24 hour incubation with Ac-LDL (50 µg of protein/ml). Medium containing Ac-LDL was removed prior to LXR agonist treatment for 24 h or 48 h.

### Microarray Procedures

Total RNA Isolation: Human total RNA was isolated with the use of Trizol (Sigma-Aldrich). RNA quality and integrity were determined using the Agilent RNA 6000 Nano Kit on the Agilent 2100 Bioanalyzer (Agilent Technologies). RNA was quantified by measuring A260 nm on the ND- 1000 Spectrophotometer (NanoDrop Technologies).

RNA Amplification and Labeling: Sample labeling was performed as detailed in the “One-Color Microarray-Based Gene Expression Analysis protocol (version 5.5, part number G4140-90040). Briefly, 1 µg of each total RNA samples was used for the amplification and labeling step using the Agilent Low RNA Input Linear Amp Kit (Agilent Technologies) in the presence of cyanine 3-CTP (Perkin Elmer). Yields of cRNA and the dye-incorporation rate were measured with the ND-1000 Spectrophotometer (NanoDrop Technologies).

Hybridization of Agilent Whole Human Genome Oligo Microarrays: The hybridization procedure was performed according to the “One-Color Microarray- Based Gene Expression Analysis protocol (version 5.5, part number G4140-90040) using the Agilent Gene Expression Hybridization Kit (Agilent Technologies). Briefly, 1.65 µg Cy3- labeled fragmented cRNA in hybridization buffer was hybridized overnight (17 hours, 65°C) to Agilent Whole Human Genome Oligo Microarrays 4x44K using Agilent's recommended hybridization chamber and oven. Following hybridization, the microarrays were washed once with 6× SSPE buffer containing 0.005% N-lauroylsarcosine for 1 min at room temperature followed by a second wash with preheated 0.06× SSPE buffer (37°C) containing 0.005% Nlauroylsarcosine for 1 min. The last washing step was performed with acetonitrile for 30 sec.

Scanning and data analysis: Fluorescence signals of the hybridized Agilent Microarrays were detected using Agilent's Microarray Scanner System (Agilent Technologies). The Agilent Feature Extraction Software (FES) was used to read out and process the microarray image files. For determination of differential gene expression FES derived output data files were further analyzed using the Rosetta Resolver gene expression data analysis system (Rosetta Biosoftware). The data discussed in this publication have been deposited in NCBI's Gene Expression Omnibus and are accessible through GEO Series accession number GSE13407 (http://www.ncbi.nlm.nih.gov/geo/query/acc.cgi?acc=GSE13407).

### Quantitative PCR analysis

Total RNA from monocytes and macrophages was extracted using Trizol (Life technologies). One hundred to 300 ng of RNA was reverse-transcribed into cDNA using M-MLV reverse transcriptase, Random Primers and RNAseOUT inhibitor (Life technologies). cDNA were quantified by real time PCR using a SYBR® Green Real-time PCR kit (Life technologies) on a LightCycler 2.0 detection system (Roche Diagnostics). Relative mRNA levels were determined using the ΔΔCt method. Values were expressed relative to cyclophilin A levels. The sequences of the oligonucleotides used are described in Table 1.

**Table 1:** Oligonucleotides used in the study.

	Sense	Antisense
CD226	5′-cggaatgcctctgaagatgat-3′	5′-cacaggccacgtcatctga-3′
CD244	5′-tgttagctgggaaagccaca-3′	5′-aagggtgccaaggaacagt-3′
CD82	5′-tggtacacaggccagtcctca-3′	5′-ggctgctgaagcaggagat-3′
Cyclophilin A	5′-gcatacgggtcctggcatcttgtcc-3′	5′-atggtgatcttcttgctggtcttgc-3′
LXRα	5′-gaagaaactgaagcggcaaga-3′	5′-actcgaagccggtcagaaaa-3′
LXRβ	5′-tgcctggtttcctgcagct-3′	5′-agatgttgatggcgatgagca-3′

### SiRNA transient transfections

Human macrophages were transfected with INTERFERin™ transfection reagent (Polyplus transfection) according to manufacturer's instructions. Briefly, 1×10^5^ monocytes were seeded in a 24-well plate and differentiated into macrophages as described above. Silencer® Select siRNA (sequences have been described previously [25]) or Negative siRNA control (Life technologies) diluted in serum-free medium were incubated with INTERFERin™ for 10 min at room temperature and added to the cells to a final concentration of 1 nM. Twenty four hours after transfection, the cells were treated before QPCR experiments.

### Flow cytometry assay

Human primary monocytes were stained with anti-CD226, CD244 or CD82 antibodies. Briefly, after *in vitro* treatment, monocytes were washed in PBS-0.1% azide and then incubated with specific antibodies or control immunoglobulin diluted in PBS-0.1% azide-0.5% BSA for 1 h at 4°C in the dark. Then after, cells were washed in PBS-azide and analysed by flow cytometry with a LSRII flow cytometer (Becton Dickinson). Human primary PBMCs were also stained as above with an anti-CD14-APC to discriminate monocytes among PBMCs.

Data were analyzed with FlowJo software (Tree Star, Ashland, Oregon).

### Statistical analysis

Mann-whitney or Wilcoxon T test were used to determine the significance between the data means.

## Supporting Information

Figure S1 **Relative proportion of CD14+ cells determined by FACS analysis after Ficoll gradient centrifugation and negative selection by using the Monocyte Isolation Kit.**
(TIF)

Figure S2 **Effect of T0901317 treatment on mRNA levels of LXR target genes.** Monocytes were treated with indicated concentrations of T0901317 at indicated times. SREBP1c, ABCA1, ABCG1 and ApoE mRNA were evaluated by quantitative PCR. Each bar is the mean ± S.D. of triplicates determination. *: significantly different from DMSO treatment (P<0.05 Mann-Whitney test).
(TIF)

Figure S3 **SREBP1c regulation by LXR α and β isoforms.** Monocytes differentiated into macrophages were transiently transfected with either non-targeted siRNA (Neg), or siRNA specific for LXRα, LXRβ, or both and then treated for 24 hours with DMSO or 10 µM T0901317. SREBP1c mRNA expression was evaluated by quantitative PCR. Each bar is the mean ± S.D. of triplicates determination. *: significantly different from DMSO treatment same siRNA conditions; +: significantly different from negative siRNA same treatment conditions (P<0.05 Mann-Whitney test).
(TIF)
