# Supplementary figures and images for: Identification of Biological Markers of Liver X Receptor (LXR) Activation at the Cell Surface of Human Monocytes
Source: PLoS One. 2012 Nov 21;7(11):e48738. doi: 10.1371/journal.pone.0048738 (PMC3504056; doi:10.1371/journal.pone.0048738)

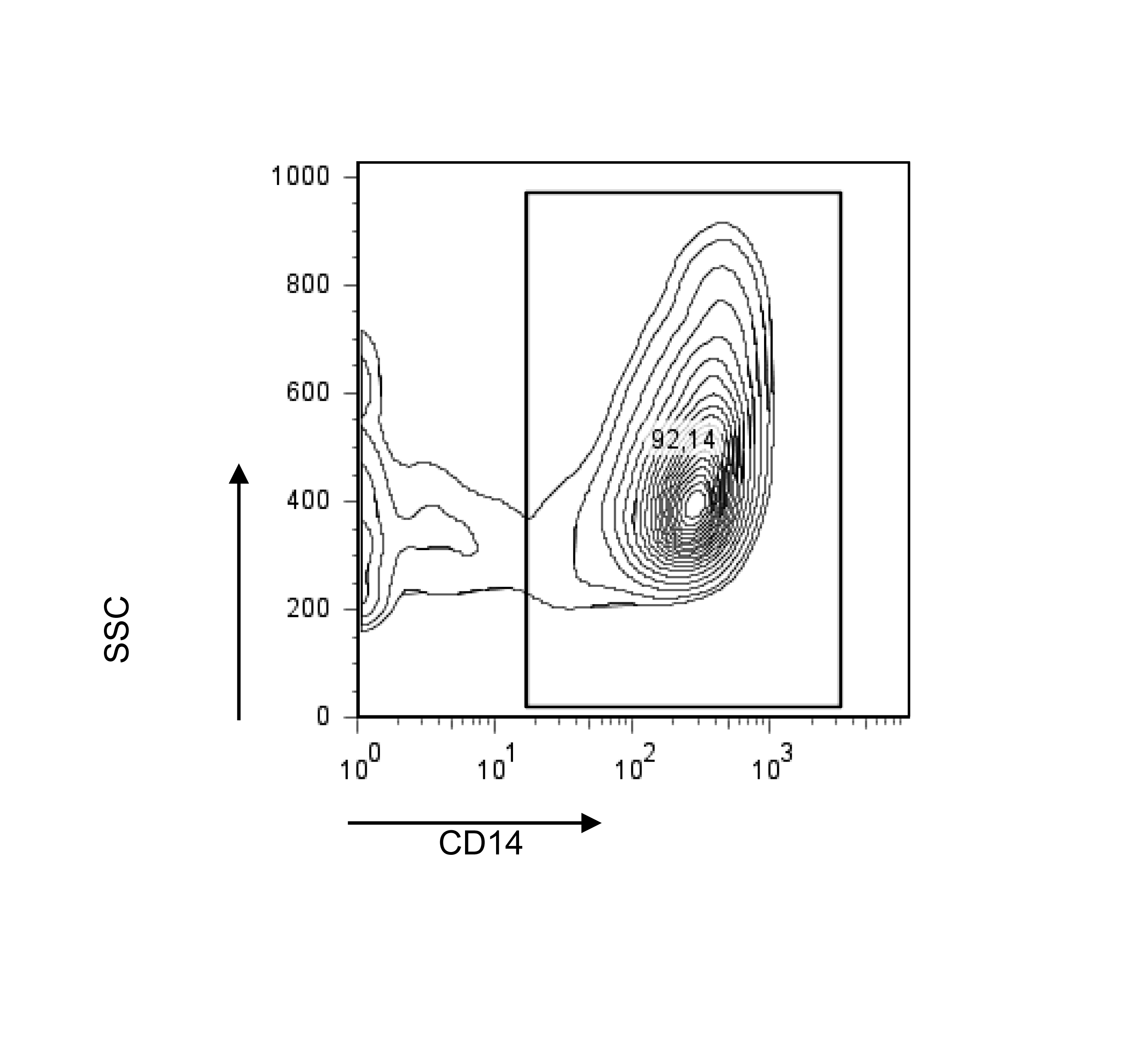

Supplement: Figure S1 — Relative proportion of CD14+ cells determined by FACS analysis after Ficoll gradient centrifugation and negative selection by using the Monocyte Isolation Kit. (TIF) [file pone.0048738.s001.tif]

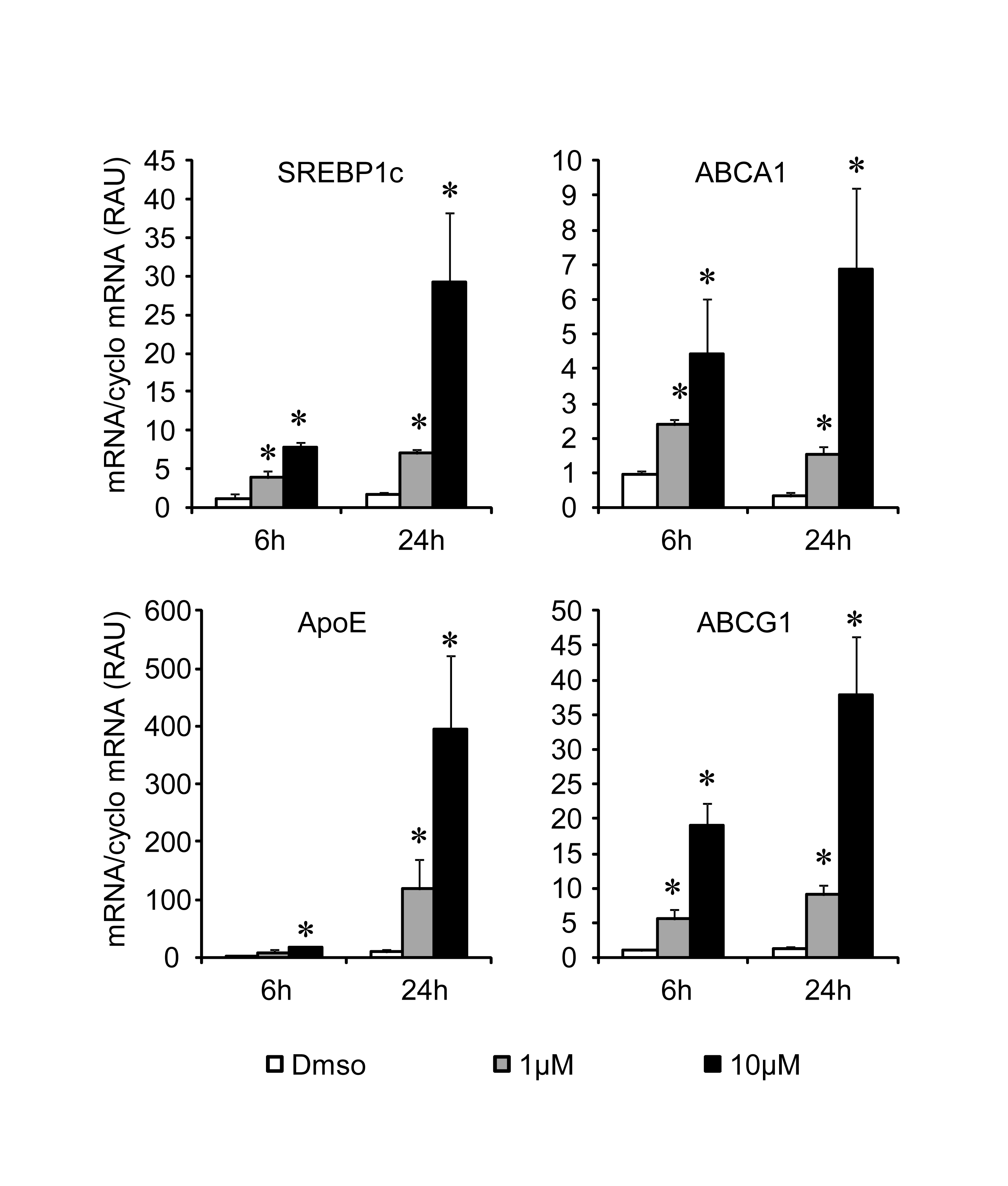

Supplement: Figure S2 — Effect of T0901317 treatment on mRNA levels of LXR target genes. Monocytes were treated with indicated concentrations of T0901317 at indicated times. SREBP1c, ABCA1, ABCG1 and ApoE mRNA were evaluated by quantitative PCR. Each bar is the mean ± S.D. of triplicates determination. *: significantly different from DMSO treatment (P<0.05 Mann-Whitney test). (TIF) [file pone.0048738.s002.tif]

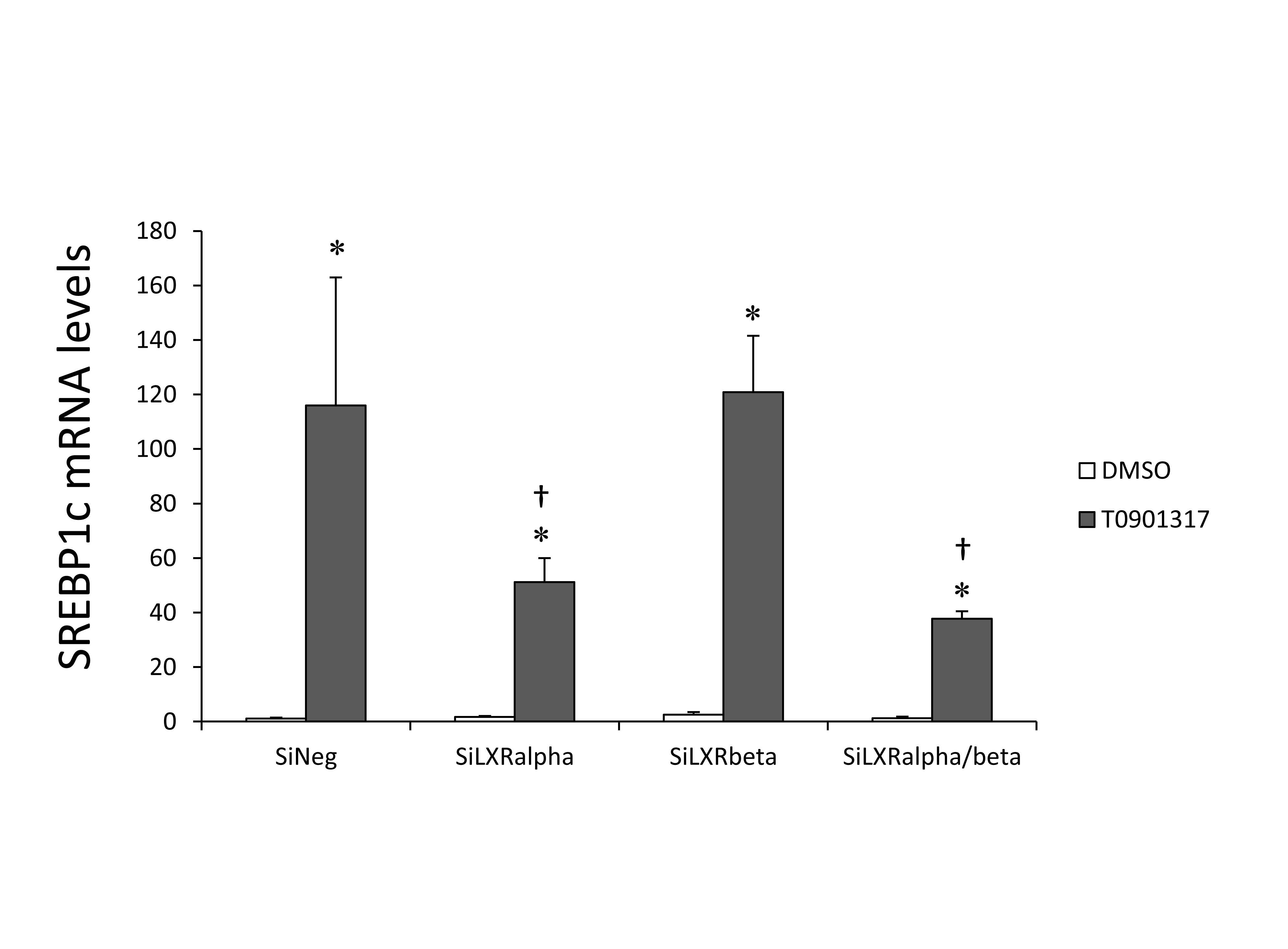

Supplement: Figure S3 — SREBP1c regulation by LXR α and β isoforms. Monocytes differentiated into macrophages were transiently transfected with either non-targeted siRNA (Neg), or siRNA specific for LXRα, LXRβ, or both and then treated for 24 hours with DMSO or 10 µM T0901317. SREBP1c mRNA expression was evaluated by quantitative PCR. Each bar is the mean ± S.D. of triplicates determination. *: significantly different from DMSO treatment same siRNA conditions; +: significantly different from negative siRNA same treatment conditions (P<0.05 Mann-Whitney test). (TIF) [file pone.0048738.s003.tif]
